# Promoting healthy weight for all young children: a mixed methods study of child and family health nurses’ perceptions of barriers and how to overcome them

**DOI:** 10.1186/s12912-020-00477-z

**Published:** 2020-09-14

**Authors:** Heilok Cheng, Rosslyn Eames-Brown, Alison Tutt, Rachel Laws, Victoria Blight, Anne McKenzie, Chris Rossiter, Karen Campbell, Kyra Sim, Cathrine Fowler, Rochelle Seabury, Elizabeth Denney-Wilson

**Affiliations:** 1 Susan Wakil School of Nursing and Midwifery, Faculty of Medicine and Health, The University of Sydney, Sydney, Australia; 2 Child and Family Health Nursing, South Western Sydney Local Health District, NSW Health, Sydney, Australia; 3 Institute of Physical Activity and Nutrition, School of Exercise and Nutrition Sciences, Faculty of Health, Deakin University, Melbourne, Australia; 4 Sydney Local Health District, NSW Health, Sydney, Australia; 5 Centre for Midwifery, Child and Family Health, School of Nursing and Midwifery, Faculty of Health, University of Technology Sydney, Sydney, Australia; 6 Centre for Population Health, NSW Ministry of Health, Sydney, Australia

**Keywords:** Pediatric obesity, Infant, Obesity management, Community health nurses, Rapid weight gain, Child and family health nurses, Cultural diversity, Health care survey, Ethnically diverse populations, Qualitative research

## Abstract

**Background:**

Childhood obesity is a global health concern. Early intervention to help parents adopt best practice for infant feeding and physical activity is critical for maintaining healthy weight. Australian governments provide universal free primary healthcare from child and family health nurses (CFHNs) to support families with children aged up to five years and to provide evidence-based advice to parents. This paper aims to examine factors influencing the child obesity prevention practices of CFHNs and to identify opportunities to support them in promoting healthy infant growth.

**Methods:**

This mixed methods study used a survey (*n* = 90) and semi-structured interviews (*n* = 20) with CFHNs working in two local health districts in Sydney, Australia. Survey data were analysed descriptively; interview transcripts were coded and analysed iteratively. Survey and interview questions examined how CFHNs addressed healthy infant feeding practices, healthy eating, active play and limiting sedentary behaviour during routine consultations; factors influencing such practices; and how CFHNs could be best supported.

**Results:**

CFHNs frequently advised parents on breastfeeding, introducing solid foods, and techniques for settling infants. They spent less time providing advice on evidence-based formula feeding practices or encouraging physical activity in young children. Although nurses frequently weighed and measured children, they did not always use growth charts to identify those at risk of becoming overweight or obese. Nurses identified several barriers to promoting healthy weight gain in infants and young children, including limited parental recognition of overweight in their children or motivation to change diet or lifestyle; socioeconomic factors (such as the cost of healthy food); and beliefs and attitudes about infant weight and the importance of breastfeeding and physical activity amongst parents and family members.

**Conclusions:**

CFHNs require further education and support for their role in promoting optimal child growth and development, especially training in behaviour change techniques to increase parents’ understanding of healthy infant weight gain. Parent information resources should be accessible and address cultural diversity. Resources should highlight the health effects of childhood overweight and obesity and emphasise the benefits of breastfeeding, appropriate formula feeding, suitable first foods, responsiveness to infant feeding cues, active play and limiting screen time.

## Background

Rates of childhood obesity are increasing worldwide [1]. In Australia, the prevalence of combined overweight and obesity in young children aged 2 to 4 increased from 4% in 1995 to 24.6% in 2017–2018 [2, 3]. Childhood obesity increases the risk of non-communicable diseases, including type 2 diabetes, cardiovascular disease, obstructive sleep apnoea, musculoskeletal conditions, gastroesophageal reflux, gallstones and non-alcoholic fatty liver disease before adulthood [1, 4]. Having overweight or obesity limits childhood development, as excess weight inhibits movement and activity levels. It may delay both motor and mental development from an early age [5, 6] with impacts sustained well into childhood [7].

Feeding practices in early infancy are critical. Obesity risk is associated with the use of infant formula instead of breastfeeding, shorter durations of breastfeeding, early or late introduction of solids (before four or after 7 months) and use of follow-up and toddler formula [1, 8–10]. Breastfeeding may protect against overweight and obesity [11, 12], through its nutritional components and because this mode of nutrition encourages self-regulation and avoids overfeeding [13]. Formula feeding may accelerate growth, as its protein composition differs from human milk and the associated parental control (e.g. more frequent feeding, bottle emptying) results in less response to infant appetite cues [14]. Early introduction of solid foods before 6 months has been associated with risk of overweight and obesity in infants, continuing to toddler and preschool age [9]. Australian government guidelines advise 6 months of exclusive breastfeeding, to protect against obesity, type 1 and type 2 diabetes, and cardiovascular disease risk factors [15].

The early onset of excess weight and the potential for long term adverse effects both suggest the need for early intervention to support healthy growth and lifestyles. Primary health care practitioners are well-placed to identify, prevent and treat overweight and obesity. In Australia, child and family health nurses (CFHNs), also referred to as maternal and child health nurses, community health nurses and child health nurses in other Australian states, offer free support to all families with children aged up to 5 years. They are registered nurses with additional postgraduate certification in child and family health nursing, and are recognised as practicing at an extended level of nursing with families with infants and young children. In 2015, there were nearly 5500 registered nurses employed principally in child and family health across Australia [16]. They provide home visits, one-on-one health centre consultations, group education, telephone support [17] and primary health care assessments [18]. Within universal child and family health services, CFHNs monitor growth development, provide psychosocial support, and promote and support breastfeeding [19, 20]. CFHNs also provide additional support for clients with complex needs, such as immigrant and refugee families or women with mental health problems [17, 21–32]. A study in Melbourne, Victoria found CFHNs were highly confident in measuring height and weight to determine growth and providing lifestyle advice on breastfeeding, healthy diet and active play, but were less confident identifying infants at risk of overweight or obesity or using behaviour change strategies to support parents [24].

The CFHN role includes identifying and managing rapid infant weight gain. A study of 16 CFHNs in Melbourne found that they felt ill-equipped to raise issues such as excess weight status, and feared offending parents arising from differing cultural perceptions on child weight or damaging the effectiveness of the parent-nurse relationship if parents were non-receptive to the topic of child weight [24]. Another study indicated that parental responses, attitudes and cultural beliefs on infants are likely to affect how nurses engage with families at increased risk of overweight and obesity risk [28]. For instance, some refugees and immigrants to Australia may perceive large infants and young children as indicative of good health or prosperity, influencing parents’ infant feeding practices. They may also be influenced by newfound ease of access to infant formula; acculturation to infant formula as a Western ‘norm’; and barriers to public breastfeeding [33–37]. Further, although CFHNs reported providing good education on nutrition and oral health, they faced challenges in addressing inappropriate feeding practices, such as cariogenic discretionary foods (e.g. dissolved biscuits or sweeteners) in bottles or on pacifiers, and prolonged bottle and formula feeding [31, 32]. This highlights the need for further support for CFHNs in educating parents from diverse backgrounds about obesity risk behaviours for infants and children.

Given that the majority of Australian families visit universal CFHN services [38], nurses have a critical role in promoting healthy infant growth and offering early intervention for rapid weight gain. Recent studies have explored CFHNs’ role in addressing infant feeding within structured primary care interventions with clients with specific needs (e.g. disadvantaged families or new parents) [39–41]. However, little is known about how CFHNs implement infant feeding advice and obesity prevention in their usual practice with general populations. This study aimed to examine the child obesity prevention practices of CFHNs in two local health districts (LHDs) in Sydney, Australia, to explore the key factors influencing their practice and to identify opportunities to enhance and support CFHNs in promoting healthy infant and child growth.

## Methods

This study used a mixed methods design involving a survey followed by qualitative interviews to further elaborate survey responses.

### Setting

The two LHDs cover a broad area of metropolitan Sydney, Australia’s largest city, ranging from inner-city suburbs to rural communities, and serving a culturally diverse population. One LHD includes rapidly growing suburbs with a high birth rate. The CFHN services in these districts provide support to families free of charge through clinics in community health centres, home visits, parenting groups and telephone contact.

Both LHDs gave ethics approval for the study.

### Recruitment

All nurses working in the two CFHN services (*n* = 156) were invited to participate in an online survey by email circulated by nurse managers, between January and March 2018. Three reminders were sent, one week apart. Submission of the survey was taken as providing informed consent. Paper surveys with reply-paid envelopes were also distributed to improve engagement in the survey process.

The survey asked nurses if they were interested in participating in a telephone interview to further discuss infant feeding.

### Data collection tools

#### Survey

The survey used an instrument that was developed for a previous study conducted by the research team [24] and based on a review of the literature and previous theoretical models. The current study aimed to investigate CFHN experiences in areas with a culturally diverse population. The survey took around 15–20 min to complete, and included questions on current CFHN practices when consulting families with young children, aged 0–5 years, in relation to obesity prevention, healthy infant feeding and physical activity (Additional file 1).

#### Interview guide

The interview guide was developed to expand on survey responses addressing the obesity prevention practices of nurses, and factors that supported or impeded these practices.

Discussion covered parental receptiveness and views on government guidelines (e.g. on breastfeeding initiation and duration, introduction of solids and limiting screen time) and other resources to promote healthy eating and activity (Additional file 2). Three members of the study team conducted the interviews by telephone, at a time most convenient for the participants. 

#### Data analysis

##### Survey analysis

IBM SPSS for Windows, Version 22.0 (2013, Armonk, NY: IBM Corp) was used to analyse survey data descriptively. In the table on the frequency of practices related to obesity prevention, data were collapsed into two categories: never to sometimes (≤50% of consultations), and often to mostly (≥51% of consultations). Confidence variables were collapsed into two categories: lower confidence (‘not at all confident’ or ‘somewhat confident’) and higher confidence (‘very confident’ or ‘extremely confident’). Data on respondent agreement were collapsed into two categories: overall disagree (‘strongly disagree’ or ‘disagree’) and overall agree (‘agree’ or ‘strongly agree’).

##### Interview analysis

Interviews were transcribed verbatim and imported into NVivo 11 for Windows (2015, Melbourne, Australia: QSR International) for data coding, sorting and retrieval. Two researchers conducted the analysis process, which included: checking the accuracy of transcripts against audio recording; developing a coding framework; coding the data into broad categories using an inductive approach guided by the research aims; resolving differences in data coding; and coding within broad categories to identify and refine common and divergent views. The findings were discussed, collated and circulated to the research team for comment.

## Results

### Sample

Ninety nurses completed surveys, representing a 58% response rate of CFHNs across both LHDs. All respondents were female, with half (51%) aged 50 years or above. In terms of experience, 19% had worked as CFHNs for less than 5 years, while 52% had over 10 years’ experience (data not presented) (Additional file 3). Interviews ceased at twenty as no new themes were emerging and we considered that data saturation was achieved. Interview times ranged from 30 min to 1 h. We did not report demographic data on the twenty interviewees to preserve confidentiality.

### Survey findings

Survey respondents reported a varying caseload, ranging from one to over 30 consultations weekly, with a mode of 10–19 consultations. Three-quarters of responses reported that routine clinical assessments for infants and children up to 5 years accounted for the majority (≥51%) of consultations; other key reasons were breastfeeding support and education on other infant feeding issues (Table 1).

**Table 1:** Proportion of nurse consultations with infants and young children, aged 0–5 years

Consultation reason	No. of responses	Proportion of all consultations - n (%)			
		None	Few (1–25%)	Some (26–50%)	Majority (≥51%)
Routine baby or child health checks	89	2 (2.2)	6 (6.7)	14 (15.7)	67 (75.3)
Breastfeeding advice or support	86	0	18 (20.9)	43 (50.0)	25 (29.0)
Other infant feeding advice or support (excluding breastfeeding)	85	0	21 (24.7)	41 (48.2)	23 (27.1)
Immunisations	80	70 (87.5)	3 (3.8)	1 (1.3)	6 (7.6)
Acute health problem	81	33 (40.7)	41 (50.6)	7 (8.6)	0
Chronic health problem	83	33 (37.5)	46 (57.5)	4 (5.0)	0

Most respondents (≥90%) had easy access to growth and body mass index (BMI) charts, and educational materials on infant feeding, infant sleep and settling, and healthy eating for pre-schoolers and toddlers. Fewer respondents could easily access education materials on promoting active play (86%) and limiting sedentary behaviours (61%) (data not presented) (Additional file 3).

Almost all respondents agreed that providing advice on infant feeding (100%) and healthy lifestyle behaviours for the whole family (95%) was central to their role. Many believed that their advice and support promoted the adoption of healthy lifestyle for the whole family (75%) and most found it professionally rewarding to address healthy lifestyle behaviours with families of young children (94%) (data not presented) (Additional file 3).

Table 2 indicates how often CFHNs addressed specific issues related to healthy weight gain.

**Table 2:** Frequency of activities provided in typical consultations with young children, aged 0–5 years

	No. of responses	Proportion of consultations, n (%)	
		Never to sometimes (≤50%)	Often to mostly (≥51%)
**Feeding advice and support**			
Encouraging continuation of breastfeeding in breastfeeding mothers	87	12 (13.8)	75 (86.2)
Offering water as the main drink for children ≥12 months	87	12 (13.8)	75 (86.2)
When to introduce solids to infants	87	14 (16.1)	73 (83.9)
How to introduce solids to infants	87	13 (14.9)	74 (85.1)
Parents eating meals with their children	87	14 (16.1)	73 (83.9)
Limiting intake of sweetened drinks	87	12 (13.8)	75 (86.2)
Increasing fruit and vegetable intake	87	27 (31.0)	60 (69.0)
Limiting high sugar and/or high fat foods	86	28 (32.6)	58 (67.4)
Provide correct formula preparation advice to parents who are formula feeding their infants	87	47 (54.0)	40 (46.0)
**Behaviour advice and support**			
Sleep and settling techniques for infants	88	13 (14.8)	75 (85.2)
Limiting TV or other screen-based activities	88	30 (34.1)	58 (65.9)
Limiting TV and electronic media use to ≤1 h/daily for children aged 2–5 years	86	33 (38.4)	53 (61.6)
Increasing active play for young children	87	36 (41.4)	51 (58.6)
**Growth charts and measurements**			
Measure height and weight of children aged ≤2 years	87	7 (8.0)	80 (92.0)
Plot height and weight of children aged ≤2 years on growth chart	87	9 (10.3)	78 (89.7)
Use growth or BMI chart to identify infant or child at risk of overweight or obesity	84	37 (44.0)	47 (56.0)
Measure height and weight of children aged ≥2 years	87	40 (46.0)	47 (54.0)
Calculate BMI of children aged ≥2 years and plot on BMI percentile chart	85	49 (57.6)	36 (42.4)
**Referral to other services**			
Referral to an allied health professional	33^a^	22 (66.6)	11 (33.3)
Referral to dietitian	87	75 (86.2)	12 (13.8)
Referral to weight management clinic	87	80 (92.0)	7 (8.0)

*BMI* body mass index.

^*a*^ *Some respondents using the paper survey did not indicate how often they referred to allied health professionals. 51 respondents reported referring to allied health, with the most common referral being to dietitians (24), speech pathologists (20), physiotherapists (15), and occupational therapists (14), as well as feeding clinics, dental services, psychologists and lactation consultants*

The issues ‘often’ or ‘mostly’ addressed by CFHNs with parents in a typical consultation pertained to breastfeeding, food and fluid intake, eating habits, sleep techniques and limiting screen time (Table 2).

The questions did not explore what CFHNs did in consultations after they calculated a child’s body mass index, although Table 2 indicates that small proportions regularly make referrals to other health services including dietitians and or weight management clinics. While CFHNs frequently measured height and weight, which is plotted on growth charts for children aged less than 2 years old (92 and 90% respectively), these activities were less frequent for children aged between 2 and 5 years (54 and 42% respectively). Just over half of the survey respondents (56%) ‘often’ or ‘mostly’ used growth or BMI charts to identify infants or young children at risk of overweight and obesity, regardless of the age of the child.

Respondents were less likely to address some aspects of nutrition: only two-thirds ‘often’ or ‘mostly’ discussed increasing fruit and vegetable consumption or limiting foods high in fats or sugar. They also advised parents on physical activity less often. It is important to note that less than half the respondents reported that they ‘often’ or ‘mostly’ advised parents on best-practice formula preparation.

Most survey respondents reported higher confidence in these activities (data not reported) (Additional file 3). Some respondents reported lower confidence in calculating the BMI of children aged 2–5 years and plotting this on a BMI percentile chart (13%), and identifying infants and young children at risk of overweight and obesity (16%).

The health professional guidelines used most commonly by respondents were the national infant feeding (93%) and healthy eating guidelines (84%), with physical activity (64%) and sedentary guidelines (51%) used less often. Other resources used by nurses included websites and brochures from health professional industry organisations [42–44] and state government bodies [45–47].

### Barriers to parental uptake of lifestyle advice for infants and children

Survey respondents identified barriers that could reduce parental uptake of lifestyle advice for infants and children, rated as moderately or very important (Table 3). Over half the respondents identified the following barriers: lack of recognition that child is overweight; lack of motivation to make lifestyle changes; lack of concern or action about child’s weight; lack of priority attached to child’s weight; and concerns that parents will not find the advice relevant or effective. The impact of socio-economic factors such as the cost of healthy food on infant and child feeding decisions was also an important barrier. Over 60% of respondents also considered that limited clinical services or insufficient time for health promotion were important structural barriers.

**Table 3:** Key barriers affecting promotion of healthy weight gain in infants and young children^a^

Barrier	N	n (%) responses rating the barrier as important^b^		
		Moderately important	Very important	Total
Parent doesn’t recognise child is overweight	86	30 (34.9)	54 (62.8)	84 (97.7)
Parent not motivated to change diet or lifestyle	86	19 (22.1)	61 (70.9)	80 (93.0)
Parent is overweight, so unconcerned that child is overweight	84	30 (35.7)	47 (56.0)	77 (91.7)
Socio-economic factors (e.g. cost of healthy food)	86	32 (37.2)	46 (53.5)	78 (90.7)
Child’s weight not a parental priority	87	39 (44.8)	29 (33.3)	68 (78.2)
Advice is not effective	84	25 (29.8)	34 (40.5)	59 (70.2)
Nurse’s concern that parents will not be receptive to advice	86	31 (36.0)	27 (31.4)	58 (67.4)
Advice irrelevant to presenting issue	85	30 (35.3)	26 (30.6)	56 (65.9)
Lack of clinical services for additional/ongoing parental support	85	28 (32.9)	26 (30.6)	54 (63.5)
Nurse’s lack of time	83	33 (39.8)	18 (21.7)	51 (61.5)
Nurse’s concern that parents will not act on advice	85	25 (29.4)	26 (30.6)	51 (60.0)

^a^ See Additional file 1 for all questions

^b^ Remaining respondents rated the barrier as ‘not important’ or ‘slightly important’

### Perceptions on healthy weight gain promotion for infants and young children

In addition to questions on barriers to promotion of healthy weight gain, respondents described their perceptions on their role in advising parents on healthy lifestyle behaviours (data not reported) (Additional file 3).

Many CFHNs (66%) agreed that some parents reacted negatively to discussion of child’s weight. Almost half (49%) felt they did not have sufficient time to properly address healthy lifestyle behaviours with families with young children. Just over a third (35%) felt uncomfortable raising the issue of infants’ and young children’s weight with parents. Few (18%) agreed that they had clients who were generally not interested in development of healthy lifestyle habits for their children.

### Addressing barriers through nurse education

Table 4 shows the areas in which respondents had received formal training (i.e. more than one hour of professional designed instruction) in the past two years. Over half of respondents received instruction about breastfeeding, introduction of solids for infants, healthy eating and active play. Less than half received instruction about obesity prevention, obesity management, or behavioural change techniques.

**Table 4:** Nurse participation rates in education in past two years (*N* = 90)

Education topic	n (%)
Breastfeeding	80 (88.9)
Introduction of solids to infants (e.g. timing, types of foods)	60 (66.7)
Healthy eating for young children (0–5 years)	57 (63.3)
Healthy infant feeding practices (e.g. eating together as a family, use of food as reward)	55 (61.1)
Active play for young children (0–5 years)	42 (46.7)
Obesity prevention in children	41 (45.6)
Limiting sedentary behaviour (e.g. TV watching) in young children (0–5 years)	38 (42.2)
Obesity management in children	31 (34.4)
Behaviour change techniques	28 (31.1)

Formula feeding education was not addressed in the survey.

Most respondents (68, 76%) were interested in additional training in promoting healthy weight gain in young children. Respondents preferred training workshops (59%) or learning through online self-study material (39%).

### Interview findings

#### CFHN perceptions of parental views about healthy weight in infants and children

Interview data confirmed and elaborated survey findings about barriers to parental uptake of healthy weight and feeding advice from CFHNs. Qualitative analysis indicated that nurses perceived that the barriers encountered related to parents’ beliefs about health, wellness, the benefits of breastfeeding and formula use, and the use of feeding to settle infant behaviour. CFHNs also highlighted factors, such as obsolete and inaccurate practices, and cultural feeding conventions, conflicting with Australian health care advice. Interviewees recounted that parents may believe that an overweight child is a ‘normal’ size; is a familial norm; or is indicative of health or wealth. Parental beliefs about the advantages of formula feeding compared to breast feeding also challenged CFHNs’ capacity to influence their child feeding practices. Nurses suggested that these beliefs were sometimes strongly influenced or endorsed by relatives and friends. In cases where parents were open to making lifestyle changes, their intentions could be challenged or even opposed by significant relatives or friends.

##### ‘Large is healthy’, ‘normal’ or a familial norm

Interviewees explained that some parents’ beliefs or norms about infant body size resulted in a difficulty recognising weight problems in their own children, even when presented with clinical evidence.

*“So, there are ideas around some of the cultures that we work with … that a fat, large baby is a healthy baby … when they actually look at a* [n overweight] *child, they’ll look at them and go, ‘but they’re normal.’ Because they’ve actually changed the way they look at them. And when you show them what a normal child looks like, they'll argue with you that they're unhealthy.”*

(*Interviewee #15*)

In the experience of CFHNs, large body size was perceived by some cultures as reflecting good health and wealth status, and tended to be highly sought by some people from culturally and linguistically diverse (CALD) groups.

*“[For] the Chinese, Indian, Vietnamese, Nepalese … because they've come probably from very poor circumstances … it's still a symbol of wealth – fat, healthy children. But they don't necessarily want them to be fat … it’s that wealth thing, that rich people use formula, rich people do this. And here they can do it [use infant formula] … [they see] that we’re giving our baby bottles.”*

(*Interviewee #8*)

CFHNs reported that some parents, who they described as overweight themselves, considered this as their family norm. CFHNs indicated that these parents were not concerned about the issue of child overweight and were less amenable to modify health behaviours.

*“[They say] ‘Oh, we're all big in our family. It's good to be a big healthy, you know, baby.’”*

*(Interviewee #2)*

*“When I’ve had [paediatric patients] where they're overweight, and I really do want them to go and see a dietitian, or talk about it, the parents would say, ‘well, you know, there's nothing wrong with me, you know, and I'm big’, or one I can remember, they owned a pastry shop, and just said, ‘that's just how we live’.”*

*(Interviewee #16)*

#### CFHN perceptions of parental beliefs about nutrition and activity

##### Breastfeeding and formula feeding

CFHNs indicated that some parents expected breastfeeding to be a pain-free, easily mastered skill. When breastfeeding experiences were contrary to their expectations, or they perceived their milk supply to be insufficient, parents opted for formula feeding instead.

*“When [breastfeeding’s] not [great], they’re very disillusioned … formula is just so easy for people to get … they’ll go, ‘Oh, I’ll just give my baby some formula, he’ll start sleeping’, or … ‘You won’t have to feed every two to three hours’, without knowing exactly what it is that formula does to the baby …*

*… they haven't been [breast] feeding well from the start … so ‘I'll offer this bottle, oh, wow, look at that, my baby is now sleeping’. And then, you just start into that cascade until the formula just becomes the normal.”*

*(Interviewee #2)*

CFHNs reported parental beliefs that formula feeding is nutritionally equivalent to breastfeeding. These beliefs were fostered by family and social influences, which could also over-rule parents’ own breastfeeding intentions.

*“The misunderstanding in some cultures that formula fed babies are just as well catered for with formula feeding, and that formula feeding is the same as breastfeeding, which is not true.”*

*(Interviewee #20)*

##### Responding to infants’ cues

CFHNs reported parents using formula feeding or early introduction of solid foods to manage fussy eating, sleep and settling issues.

*“[Parents] think that [infants] should just be all calm and settled all the time. … So, looking at things like … baby cues, and whether they’re hungry, whether they’re tired … because a lot of them misrepresent it and they tend to [think] ‘Oh, I’ll just feed them anyways’.”*

*(Interviewee #15)*

*“ … someone has said to them, ‘Oh no, you know, if they’re not sleeping well’, or something like that, ‘oh, you need to start solids’. … But there’s always someone telling them if they’re having difficulties, ‘oh, look, you can just put the baby on the bottle’.”*

*(Interviewee #1)*

##### Family influences

Family members, especially grandparents, are often in a powerful position, directly or indirectly, to influence parents’ infant feeding practices. Difficulties arose when older, influential people promulgate outdated information, culturally traditional feeding advice, or preference for formula feeding to breastfeeding that conflicted with the evidence-based advice provided by CFHNs.

*“So, you might have a mum who’s doing really well, exclusively breastfeeding. She goes home to country and she comes back and she’s giving them a bit of both [breastfeeding and formula feeding], ‘cause that’s what [her] mum did… even though you’ve put [exclusive breastfeeding] into motion, shared it, talked to them about it, family has a really big impact on their decisions that they make.”*

*(Interviewee #5)*

*“We have a lot of Bangladeshi families coming through, and they seem to do a lot of force feeding, or hand feeding the child, and that’s a very cultural thing. So… we do a lot of talk around letting the child feed themselves, sitting with the family and eating as the family. It seems to be this thing of, you know, just trying to get the food in the child, and as much as you can, of it. … Dealing with their cultural beliefs about eating.”*

*(Interviewee #4)*

*“I think the more vulnerable people are… not as receptive and they tend to follow their families… I do see quite a few Aboriginal families, and they would tend to just follow… what's been done previously… feeding the wrong foods at the wrong times and giving too much from a bottle…”*

*(Interviewee #14)*

##### Infant physical activity and play

Interviewees identified other cultural norms that prevented parental uptake of CFHNs’ advice on healthy eating and physical activity.

*“Well, we talk to them about not using walkers at all. Unfortunately, in the Bengali culture, having a walker is being seen as wealthy. We talk about walkers as being unsafe and the fact that they actually inhibit their gross motor development rather than enhance it. … They pass them on to each other because they're seen as a sign of wealth.”*

*(Interviewee #15)*

*“I’ve revisited families and I find the more I can role model it—so if I’m talking about tummy time, if I actually show them tummy time… you can talk to it ‘til you’re blue in the face, but… in a lot of cultural situations, they don’t put their babies down on the floor.”*

*(Interviewee #4)*

##### Additional resources

Some CFHNs felt that limited clinical time and resources impeded their ability to advise parents effectively:

*“I don't know whether they necessarily have enough contact with us to appreciate sometimes what we're trying to say. I don't know whether we're making any great changes or having any great influence on their decisions… Probably because the amount of time that we get to spend with people.”*

*(Interviewee #3)*

Professional education can maintain and refresh CFHNs’ knowledge of evidenced-based practice. Almost half the interviewees reported having sufficient confidence in their practice and needed no further resources to help address healthy weight gain. Others, although confident, were interested in attending more education sessions out of professional interest.

*“I've been doing this for a long time, so I just like to do ongoing education, keep up to date with the latest guidelines, with education on difficult things, you know, certain allergies and stuff like that.”*

*(Interviewee #8)*

CFHNs who wanted to improve their confidence suggested additional topics of interest, including using behaviour change communication and techniques for parent education; motivational interviewing; anticipatory guidance and counselling to address parental resistance to change and sensitivity regarding child weight; feeding, eating and activity strategies, such as management of feeding difficulties, fussy eating, food refusal, bottle cessation, weaning from breastfeeding; and addressing cultural practices, beliefs and norms of CALD groups in the client population, particularly East Asian, South Asian, Arabic and Australian Aboriginal and Torres Strait Islander groups.

#### Clinical resources for parents

Nurses discussed the need for resources for parents and caregivers to supplement nurse education to parents. Topics included evidence-based information and resources explaining consequences of early screen exposure for infants; benefits of active play and activity; consequences of rapid weight gain, overweight and obesity on infant and child health outcomes; and health effects of breastfeeding compared to formula feeding.

*“… in terms of infant feeding, maybe [demonstrating] the effects of introducing formula to babies, so that people are aware that, ‘okay, yes, it’s an infant food, but it should only be used if there’s no other option’… what effect it is going to have on the baby…”*

*(Interviewee #2)*

*“We quite often get the question… how much TV should they watch or how much screen time should they have? … and really, the only information that we have to tell them is that the less, the better.”*

*(Interview #13)*

CFHNs suggested video resources, focused on infant food preparation and storage; positive examples of parent-child interactions for feeding, self-feeding and baby-led weaning; and examples of movement, play and physical activities for infants.

*“I would love to have some videos of… happy baby eating, feeding, playing with their food… compared to the force-fed baby…”*

*(Interviewee #6)*

They identified the need for cultural-specific resources for parents, featuring cultural foods, multilingual resources for non-English speaking family members or caregivers, and plain language and visual resources for low-English literacy clients.

*“… most of our clients are Bengali, and we didn’t have… a chart to transition them from puree food into finger foods or family foods. … the charts we did have very much… Australian foods and things like that. … So, I think the resources are very much lacking in, you know, information about their culture and what they eat, so we can address it from their point of view…”*

*(Interviewee #15)*

Resources to support families were also needed, such as infant and early childhood recipes from reliable resources; basic recipes for clients with low food literacy; parenting resources such as play equipment and toys for low-income clients; and establishment of structured health promotion programs for young children up to five years of age, similar to preventive health programs for school-aged children [47].

*“… to exercise floor time and tummy time, on the floor, I often go in, and if a family can’t buy their or provide their mats [for infant exercise], then I’ll give rubber mats or yoga mats.”*

*(Interviewee #6)*

Interviewees also suggested that information given to parents should be more positive and aim to correct parents’ awareness of physical activity levels for children and benefits of limiting screen time, independent of weight issues.

*“But with lots of parents, we do see stick their kids in front of the TV, to distract them, and to get their… housework done, and things like that. So, we try and incidentally make comments, you know, like, ‘ah, yes, it’s good that baby’s got great eye contact, but she shouldn’t be watching TV and it’s not good for their eyes, their development’. Usually, if you put it in, like, their development of their eyes, they tend to listen more, like it’s going to affect their brain.”*

*(Interviewee #12)*

Nurses discussed the merits of electronic resources (websites or apps) for parent education, particularly the need for these to be approved by health care practitioners and the difficulty of encouraging parents to use recommended sites.

*“We have very strict guidelines—they go through our clinical quality meeting. … We do have, I think, three apps on our recommended list at this time.”*

*(Interviewee #1)*

Nurses were concerned about the cost of paid apps or in-app purchases, and internet access for low-income families. One interviewee highlighted the irony of recommending web-based resources:

*“I suppose my only concern is that when we're sort of encouraging parents to use websites and apps, we're sort of condoning and using their own phone and their own devices. And then we're telling them on the other hand, to stop using their devices and pay attention to their children.”*

*(Interviewee #3)*

## Discussion

Australian research shows that parents frequently attend CFHN services for well-child checks. CFHNs are the preferred provider for advice on healthy diet and nutrition, breastfeeding support, sleep and settling issues, and information about play to support development [38]. This highlights their central public health role in promoting optimal infant growth and development, and their credibility with many parents.

This study examined the current child obesity prevention practices of CFHNs in two LHDs in Sydney, Australia, and the factors influencing them. Similar to a study in Melbourne [24], these CFHNs frequently undertake growth monitoring, use evidence-based resources, address infant feeding practices, and provide infant sleep and settling techniques important in obesity prevention. However, they less frequently use BMI or growth charts to identify children at risk of overweight and obesity or provide advice on correct formula preparation for parents using formula feeding.

Some findings on the frequency of specific activities in CFHNs’ practice may relate to the profile of children attending CFHN visits. The questions on the frequency of undertaking activities (Table 2) asked nurses to indicate them as a proportion of all consultations for children aged 0–5 years, not as a proportion of clients in a specific age range (i.e. 0–2 years and 2–5 years). Parents are less likely to visit CFHNs as their children grow older [38, 48] and some CFHN programs in the participating LHDs are specifically targeted to infants up to 2 years.

Subsequently, the lower reported frequency of growth monitoring for children aged over 2 years, or discussing options for active play and limited screen time for young children, may be explained by nurses who spend the majority of their time working with infants aged under 2 years. However, even if these practices are less frequent for children over two, these factors are potentially related to excess weight gain. It is therefore vital that CFHNs have appropriate education and resources to conduct them effectively whenever necessary.

Results indicated that frequency of height and weight measurement, then calculating and plotting BMI on charts for children aged 2–5 years, was lower than the equivalent monitoring on growth charts for infants aged 0–2 years. Nurse authors from the participating LHDs advised that BMI was automatically calculated and plotted after child height and weight was entered into electronic health record software. It is unclear why respondent-reported frequency of these activities undertaken in typical consultations with young children was low. Potential reasons may be that the primary reason for a consultation in infants, aged 0–2 years, is to monitor height and weight, whereas for children, aged 2–5 years, the primary reasons for consultation relate to development, behavioural issues, speech issues, and toilet training. Further, these consultations may take place outside a personal health record check, and if no parental concerns are voiced on height and weight, clinicians will focus on priorities parents articulate.

Responses indicate that the nurses are working with, at times, limited consultation time and health promotion resources to support families of young children and build strong relationships. Through their specialist CFHN education, they have an in-depth understanding of infant feeding and growth, enabling them to offer advice and support at most visits [49]. However, CFHNs identified key barriers to promoting healthy weight in infants and children that centred on parental behaviour and attitudes, including parents not recognising child overweight status, not being motivated to change lifestyle or diet, or not being concerned about child overweight if they are, themselves overweight. Interviews with CFHNs expanded on these topics and identified the importance of parental beliefs about healthy body size and weight, limited understanding on benefits of breastfeeding compared to formula feeding, and cultural perspectives on weight and feeding behaviour.

Findings on the cultural barriers to promotion of breastfeeding and healthy weight gain in children and infants are consistent with that in previous literature, including: pressure to fully or partially replace breastfeeding with infant formula [37, 50–54]; use of formula to encourage infant sleeping, feeding by other family members or reduce infant crying [36, 37, 50, 51, 55–58]; beliefs that large infants signified health, especially from countries where infants at are risk of malnutrition and undernutrition [36, 50, 52, 57–63]; limited access to support from nurses and midwives for infant feeding and care [54, 55, 64, 65]; and introduction of solid foods before 4–6 months [33, 56, 66], across South Asian, East Asian, Middle Eastern, African, Maori, Pacific Islander and Indigenous Australian populations and peoples migrating to overseas countries. Specific cultural beliefs were also identified, such as forceful infant feeding for weight gain by Bangladeshi parents, from fear that inadequate nutrition would result in child sickness or death [59, 67, 68], or norms where playing with children may not be common practice [69, 70].

CFHNs also reported lack of appropriate resources such as resources not tailored to cultural and religious groups [56, 71], and lack of services or resources in community languages or simple English [33, 61, 71, 72]. Selection and development of culturally appropriate resources may be difficult, as it requires use of appropriate language; targeting the client’s literacy level; specification to cultural and religious backgrounds and habits; and development with local community members [73]. Resources developed by Australian national and state government health bodies have been identified to contain culturally inappropriate information, such as multilingual translations containing unfamiliar or inappropriate concepts; unintended messages about cultural norms; lack of specificity for cultural practices [73].

CFHNs in this study reported high confidence in providing advice about introducing solid foods and healthy eating behaviours, although some were less confident in growth monitoring and identifying risk of overweight and obesity. However, previous research has shown that Australian parents require more education on evidence-based introduction of solids, to increase their understanding on infants’ readiness and to counter inaccurate guidance from family members or commercial baby food packaging [74]. Another study on Australian parents with children aged 2–5 years reported that CFHNs ‘brushed over’ their child’s weight issues and offered limited advice [75].

This research found that promoting healthy growth and avoiding rapid or excess weight gain can be challenging in families from cultural groups who may value formula feeding or large body size as symbols of health and status. Our findings identified not only parental negativity or defensiveness in discussing children’s weight, but also nurses’ own sense of discomfort. Growth and BMI charts are essential for CFHNs to calculate the risk of overweight or obesity, and to illustrate those risks to parents in a factual and non-stigmatising manner focused on health rather than fatness. Nurses identified that parental receptiveness to advice was frequently a barrier to promoting healthy weight gain.

Given that few nurses had formal recent training on behaviour change techniques (Table 4), nurses may benefit from additional training and support to engage parents from all cultural backgrounds, and to initiate ‘difficult’ conversations. Teaching nurses more about behavioural change techniques could assist them in advancing parents’ knowledge of strategies to maintain healthy weight among infants and young children, and equip them to support parental decisions and behaviour change.

## Limitations

This study was limited in scope due to its setting in two LHDs and the findings may not be applicable elsewhere in Australia. The sample was self-selected, potentially skewing responses to those who were more confident or engaged in this topic. Further, respondents reported on their own practice and confidence, without alternative evidence to verify whether health promotion activities are undertaken or their effectiveness.

The survey questionnaire was not a validated instrument, although it had been used previously with CFHNs in another jurisdiction [24]. The question on CFHNs’ participation in professional education did not include training on formula feeding.

## Conclusions

This research found that nurses are providing professional support to families of young children and build strong relationships. They are experts in infant feeding and growth, and offer advice and support at most visits.

Numerous training opportunities are available to nurses, and in some cases, the burden of completing all of the required and optional training is cumbersome [76]. A key recommendation of this research is that an audit be conducted of available training and that it be consolidated and updated to ensure it is engaging and offered on the best possible online learning platform using adult learning pedagogy.

Nurses support families from many cultural backgrounds and this research found that supporting healthy growth and avoiding rapid or excess weight gain can be challenging in families from cultural groups who may value large body sizes. This study recommends that nurses are supported to provide culturally appropriate support to these families using resources available in community languages. As health literacy is sometimes limited, we recommend the development of written and visual cultural resources for use online or in a smartphone app. We recommend development of an app embedded with videos covering best practices in infant feeding, provided to all parents in the antenatal or early postnatal period.

A final recommendation is that the identification, causes and consequences of rapid weight gain be included in in-service or workshop education in a form where nurses can observe, then practise consultations to address rapid weight gain. This will increase self-efficacy in raising this sensitive issue. The results of this survey have contributed to the introduction of overweight and obesity action plans in both participating LHDs, and the inclusion of new state government resources and guidelines on childhood healthy weight management as a clinician resource [77].

## Supplementary information

**Additional file 1.**

**Additional file 2.**

**Additional file 3.**

## Data Availability

Summary data or data analysed during the current study are available from the corresponding author on reasonable request.

## Abbreviations

BMI: Body mass index
CALD: Culturally and linguistically diverse
CFHN: Child and family health nurse/nursing
LHD: Local health district
