# Supplementary material for: Promoting healthy weight for all young children: a mixed methods study of child and family health nurses’ perceptions of barriers and how to overcome them
Source: BMC Nurs. 2020 Sep 14;19:84. doi: 10.1186/s12912-020-00477-z (PMC7488672; doi:10.1186/s12912-020-00477-z)
Supplement: Supplementary file 1 — Additional file 1. [file 12912_2020_477_MOESM1_ESM.pdf]

[redacted]

Child Family Health Nurse (C&FHN) Survey

Exploring the infant feeding advice provided by Child & Family Health Nurses in [redacted]

PLEASE TICK BOX TO CONFIRM YOU HAVE NOT ALREADY COMPLETED THIS SURVEY ON-LINE

☐

This survey takes approximately 10-15 minutes to complete. All information that you provide will be kept **confidential** and stored securely. This survey is for research purposes only, it will be de-identified, your name will not be known and your individual results will not be reported.

1. Approximately how many consultations would you have with infants and young children (0-5 years) in a typical week ?: ____________

2. Approximately what proportion of the consultations with infants and young children (0-5 years), are for the following reason?:

	None	A few (1-25%)	Some (26-50%)	Majority (51-75%)	Almost all (>75%)
**a.** Routine baby or child health checks	○	○	○	○	○
**b.** Immunisations	○	○	○	○	○
**c.** Breastfeeding advice or support	○	○	○	○	○
**d.** Other infant feeding advice or support (excluding breastfeeding)	○	○	○	○	○
**e.** Acute health problem	○	○	○	○	○
**f.** Chronic health problem	○	○	○	○	○
**g.** Other, please specify: ______________________	○	○	○	○	○

3. Do you have easy access to the following (please tick all that apply):

**a** Standard growth charts for infants 0-2 years	○
**b.** BMI percentile chart for children aged 2-18 years	○
**c.** Education materials for parents on infant feeding	○
**d.** Education materials for parents on healthy eating for toddlers and pre-schoolers	○
**e.** Education materials for parents on promoting active play in young children	○
**f.** Education materials for parents on limiting sedentary activities (eg TV watching)	○
**g.** Education materials for parents on sleep and settling techniques for infants	○
**h.** None of the above	○

4. As part of a typical consultation with young children(0-5 years), about how often would you undertake the following activities. (Please *tick one response for each statement*).

% of Consultations with young children	Never<br>0%	Rarely<br>1-25%	Sometimes<br>26-50%	Often<br>51-75%	Most of time<br>>75%
**a.** Measure height and weight of children under 2 years	○	○	○	○	○
**b.** Plot height & weight on growth chart (for children under 2 years)	○	○	○	○	○
**c.** Measure height and weight for children over 2 years	○	○	○	○	○
**d.** Calculate body mass index (BMI) for children over 2 years and plot on a BMI percentile chart	○	○	○	○	○
**e.** Use growth or BMI charts to identify infants/children who are at risk of overweight or obesity	○	○	○	○	○
**f.** Provide advice or support to encourage continuation of breastfeeding in breastfeeding mothers	○	○	○	○	○
**g.** Provide advice on correct formula preparation to parents who are formula feeding their infants	○	○	○	○	○
**h.** Provide advice on sleep and settling techniques for infants	○	○	○	○	○
**i** Provide advice on when to introduce solid foods to infants	○	○	○	○	○
**j.** Provide advice on how to introduce solid foods to infants	○	○	○	○	○
**Provide advice about what foods to introduce to infants**					
**k.** Talk to parents about eating their meals with their children	○	○	○	○	○
**l.** Talk to parents about limiting children's intake of sweetened drinks (eg juice and soft drinks)	○	○	○	○	○
**m.** Talk to parents about offering water as the child's main drink (after 12 months of age)	○	○	○	○	○
**Talk to parents about appropriate portion sizes for their child**					
**n.** Talk to parents about limiting TV or other screen based activities	○	○	○	○	○
**o.** Talk to parents about limiting TV viewing &other electronic media use in children 2-5 years of age to less than one hour per day	○	○	○	○	○
**p.** Talk to parents about increasing active play for young children	○	○	○	○	○
**q.** Talk to parents about increasing their children's fruit & vegetable intake	○	○	○	○	○
**r.** Talk to parents about limiting high sugar and/or high fat foods (eg cakes, biscuits, lollies, chips, take away foods etc)	○	○	○	○	○
**Referral to other services**					
**s.** Refer or facilitate a referral to a dietitian	○	○	○	○	○
**t.** Refer or facilitate a referral to a weight management clinic	○	○	○	○	○
**u.** Refer or facilitate a referral to allied health professional, please state ____________________	○	○	○	○	○

5. The following statements ask about <u>your views</u> on infant feeding and TV watching in infants & young children.

Please indicate how much you agree or disagree with the statements by *<u>ticking one response for each statement</u>*.

	Strongly Disagree	Disagree	Agree	Strongly Agree
**a.** Parents should offer an alternative food if their child doesn't eat the food offered	○	○	○	○
**b.** Parents should encourage their children to eat all the food on their plate	○	○	○	○
**c.** If a parent continues to offer foods their baby hasn't previous enjoyed, they will come to enjoy them	○	○	○	○
**d**. The best way to settle a crying infant is to feed him/her	○	○	○	○
**e.** An infant under 6 months sometimes needs more than breast milk or formula to be full	○	○	○	○
**f.** An infant knows when s/he is full	○	○	○	○
**g.** TV is educational for infants and young children	○	○	○	○
**h**. Children under 2 should be allowed to watch TV	○	○	○	○
**i**.The recommendation to limit TV watching and the use of other electronic media (DVDs, computers etc) to less than one hour per day for children 2-5 years is unrealistic/unhelpful for most parents	○	○	○	○

6. How confident are you in undertaking the following (Please *tick one response for each statement*)

	Not at all confident	Somewhat confident	Very confident	Extremely confident
**a.** Measuring infants' height & weight & plotting on a growth chart	○	○	○	○
**b.** Calculating BMI for children 2 years & older & plotting on a BMI percentile chart	○	○	○	○
**c.** Identifying infants and young children who are at risk of overweight or obesity	○	○	○	○
**d.** Providing breastfeeding advice and support	○	○	○	○
**e.** Providing advice on correct formula preparation	○	○	○	○
**f.** Providing advice on sleeping and settling techniques for infants	○	○	○	○
**g.** Providing advice to parents regarding <u>when</u> to introduce solid foods to infants	○	○	○	○
**h.** Providing advice to parents regarding <u>how</u> to introduce solid foods to infants	○	○	○	○
**Providing advice on portion sizes for young children**				
**i.** Talking to parents about eating their meals with their children	○	○	○	○
**j**. Talking to parents about limiting children's intake of sweetened drinks (eg juice and soft drinks)	○	○	○	○
**k.** Talking to parents about offering water as the child's main drink (after 12 months of age)	○	○	○	○
**l.** Talking to parents about limiting TV or other screen based activities	○	○	○	○
**m.** Talking to parents about increasing active play for young children	○	○	○	○
**n.** Talking to parents about increasing their children's fruit and vegetable intake	○	○	○	○
**o**. Talking to parents about limiting children's intake of high sugar and/or high fat foods (eg cakes, biscuits, lollies, chips, take away foods etc)	○	○	○	○

7. Do you use any published guidelines (eg from government or health bodies) to inform your practice and advice regarding (please tick all that apply):

**a** Infant feeding	○
**b.** healthy eating in young children (0-5 years)	○
**c.** Physical activity in young children (0-5 years)	○
**d.** Sedentary behavior (eg TV watching) in young children (0-5 years)	○
**e.** None of the above	○

7b. If YES, please specify which guideline(s) you use:

______________________________ ______________________________

______________________________ ______________________________

______________________________ ______________________________

8. **The following statements ask about <u>your views</u> on promoting healthy weight gain in infants and young children.**

Please indicate how much you agree or disagree with the statements by *<u>ticking one response for each statement</u>*.

	Strongly Disagree	Disagree	Agree	Strongly Agree
**a.** Fatness or accelerated weight gain in infancy is NOT related to the development of overweight in childhood	○	○	○	○
**b.** It is easy to identify overweight infants and young children just by looking at them	○	○	○	○
**c.** It is easy to identify infants and young children who <u>are at risk</u> of becoming overweight	○	○	○	○
**d.** Providing advice to parents on infant feeding is an important part of my role	○	○	○	○
**e.** Talking to parents about healthy infant feeding practices fits easily into my routine consultations	○	○	○	○
**f.** I feel uncomfortable raising the issue of infants' and young children's weight with parents	○	○	○	○
**g.** For most parents, my advice & support does little to promote the adoption of a healthy lifestyle for the whole family	○	○	○	○
**h.** Providing advice on healthy eating and physical activity for the whole family is valued as an important part of my role by my managers	○	○	○	○
**i.** Some parents react negatively to me raising the issues of their child's weight	○	○	○	○
**j.** I have sufficient time to properly address healthy lifestyle behaviors with families with young children	○	○	○	○
**k.** Providing advice on healthy lifestyle behaviours for the whole family is an important part of my role	○	○	○	○
**l.** Parents I see are generally not interested in discussing the development of healthy lifestyle habits for their children	○	○	○	○
**m.** I find it professionally rewarding to address healthy lifestyle behaviours with families of young children	○	○	○	○

9. **Rate the importance of the following <u>barriers</u> to promoting healthy weight gain in infants &young children**

	Not very important	Somewhat important	Moderately important	Very important
**a.** Parent is not motivated to change diet or lifestyle	○	○	○	○
**b.** Parent is overweight, so they are not concerned that the child is overweight	○	○	○	○
**c.** Socio-economic factors affect the ability of families to make a change (eg cost of healthy food)	○	○	○	○
**d.** Lack of support for me to undertake this work in my role	○	○	○	○
**e.** My own lack of time	○	○	○	○
**f.** Parents don't recognise their child is overweight	○	○	○	○
**g.** A lack of appropriate education materials for parents available in my clinic/practice	○	○	○	○
**h.** A lack of relevance to the parent or child's presenting issue	○	○	○	○
**i.** A lack of referral pathways to provide additional/ongoing support for parents if required	○	○	○	○
**j.** A lack of clinical services to provide additional/ongoing support for parents if required	○	○	○	○
**k.** My concern that parents will not be receptive to my advice on healthy eating and physical activity	○	○	○	○
**l.** I lack confidence to counsel parents about healthy eating and physical activity	○	○	○	○
**m.** Addressing the child's weight is not a priority for parents	○	○	○	○
**n.** I lack knowledge about preventive care for child obesity (including healthy eating &physical activity recommendations)	○	○	○	○
**o.** My concern that parents will not act on my advice about healthy eating and physical activity	○	○	○	○
**p.** Advice regarding healthy eating, physical activity and reducing sedentary behavior (eg TV watching is not effective in preventing children from becoming overweight	○	○	○	○
**q.** Lack of relevance to my role	○	○	○	○
**r.** My lack of interest in promoting healthy weight gain in children	○	○	○	○
**s.** My own lifestyle habits	○	○	○	○
**t.** Other, please specify:______________________	○	○	○	○

10. **In the past 2 years have you had formal training (ie. more than one hour of professional designed instruction) on any of the following (please tick all that apply):**

**a.**	Breastfeeding	○
**b.**	Introduction of solids to infants (eg timing, types of foods)	○
**c.**	Healthy infant feeding practices (eg eating together as a family, use of food as a reward)	○
**d.**	Healthy eating for young children (0-5 years)	○
**e.**	Active play for young children (0-5 years)	○
**f.**	Limiting sedentary behavior (eg TV watching) in young children (0-5 years)	○
**g.**	Obesity prevention in children	○
**h.**	Obesity management in children	○
**i.**	Behaviour change techniques	○
**j.**	None of the above	○

11. Would you be interested in additional training in the area of promoting healthy weight gain in young children?

Yes ○

No ○

11a. If YES, please specify the main topic areas you would like further training on:

______________________________ ______________________________

______________________________ ______________________________

12. If you would like training, in what format would you like to receive it? (please tick all that apply)

**a.**	Workshop	○
**b.**	Self-study material (eg online module)	○
**c.**	Clinical supervision/mentoring	○
**d.**	Other, please specify: ____________________________	○

13. Are you?

Male	○
Female	○

14. What is your age?

20-29 years	O
30-39 years	O
40-49 years	O
50-59 years	O
60+ years	O

15. How many years have you worked as a Family and Child Health Nurse?

Less than 5 years	O
5-10 years	O
11-15 years	O
More than 15 years	O

16. Do you work

Full time	O
Part time ________ hours per week	O

17. Would you be interested in participating in an interview with a researcher to talk further about your experiences in promoting healthy lifestyle in families with young children (this will take approximately 30 minutes)

Yes O

No O

If you answered yes, please provide your details below (please note this page will be detached from the rest of your survey responses):

Name____

Best daytime contact number: ________________________________

Email address:____

Names of the main suburbs in which you work: ____

END OF SURVEY

THANK YOU FOR YOUR PARTICIPATION.
