# Supplementary material for: Promoting healthy weight for all young children: a mixed methods study of child and family health nurses’ perceptions of barriers and how to overcome them
Source: BMC Nurs. 2020 Sep 14;19:84. doi: 10.1186/s12912-020-00477-z (PMC7488672; doi:10.1186/s12912-020-00477-z)
Supplement: Supplementary file 2 — Additional file 2. [file 12912_2020_477_MOESM2_ESM.docx]

**Exploring the Infant Feeding Advice provided by Child & Family Health Nurses**

**Qualitative Interview Schedule**

# 1. Role

1. To start out discussion, in a few sentences, can you tell me a little bit about the focus of your role and the type of work you do?

***Healthy eating/active play advice***

# 2. Opportunities, Barriers and Motivations

*Now moving deeper into the topic, we know that CFHNs provide parents with advice and support on infant feeding, healthy eating and active play.*

1. How much of an opportunity do you actually have in practice, to talk to parents about infant feeding, healthy eating and active play? (Prompt: how much of this happens opportunistically as issues arise versus providing routine advice as per ages and stages schedule?)

*Given your workload and everything else you need to do:*

1. How much of a priority is it for you to address healthy eating and active play in the infants and young children that you see? (Prompt: do you see it as part of your role?)
2. What barriers if any, have you encountered, in providing this type of advice or support? (Prompts: time, parental receptiveness, your role, types of families you see)
3. What could be done to overcome or to more effectively manage these barriers?

# 3. Perceptions of parental receptiveness to advice

*Now, turning to your perceptions of parental receptiveness…*

1. How open do parents tend to be to the infant feeding, healthy eating and active play advice you provide?
2. What factors, if any, seem associated with parental receptiveness (or lack of it) to your suggestions and advice? (Prompt: cultural background, SES, parent overweightness, motivators, de-motivators)
3. How effective is your advice/support in influencing parents’ decisions about infant feeding, healthy eating and active play for their child?

# 4. Ways to support the CFHN role

*The pooled survey results show that nurses usually have areas of practice in which they would like to feel more confident.*

1. How well equipped (supported) do you feel, in terms of information, collegial support, other resources etc., to address healthy lifestyle behaviours in the families that you see?
2. What can you tell me about your main sources of information/knowledge about infant feeding, healthy eating and active play in young children? (e.g. who provides it? Is it useful?)
3. What, if anything, might help to boost your confidence in addressing healthy lifestyle behaviours with the families?
4. What, if any, additional support might be helpful to you?

# 5. Perceptions of current government guidelines on key behaviours.

## 5.1. Breastfeeding/formula feeding

*I would now like to talk a little bit about your experiences of promoting breastfeeding.*

1. The current government recommendation is that mothers exclusively breastfeed their infant to 6 months of age. What do you think about this? (Prompts: why do you think this is important? Do you discuss the recommendation with mothers?)
2. From your experience, is it harder for mothers in some socio-economic groups to start and continue with breast feeding their infant? (Prompts: which group/s find it hardest? What are the main barriers?)
3. How and when, if at all, might mothers’ breast feeding decisions be influenced?
4. When you talk to mothers who have chosen to formula feed, what sorts of issues do you cover?
5. What would help you to support mothers to breastfeed their babies?

## 5.2. Introduction of solids

*I would now like to talk a little bit about providing advice on the introduction of solid foods.*

1. The government recommendation is that solid food should be introduced at around 6 months of age. What is your view about the recommendation?

If AGREE 🡪 Why do you think this is important? Is this something you discuss with mums?

If DISAGREE 🡪 What do you use as your guide in deciding when solid food should be introduced? What are your views about the timing of the introduction of solids?

1. What information do you discuss with mothers that are starting to introduce solids? (Prompts: types of food, repeated exposure to food to promote acceptance, when and how much to feed their baby).
2. What would help you in providing advice or support to mums around the introduction of solids (e.g. clear information about reason for delaying solids, video of how to prepare foods, etc.)

## 5.3. TV watching/screen use

*I would now like to ask your views about TV watching and encouraging active play in young children.*

1. What do you think about the recommendation that children under 2 years should not watch any TV? Do you think this is realistic for most parents?
2. How do you feel about making suggestions to parents to limit TV and other media such as DVDs, iPads etc.? Is this something that you do? How do parents respond?
3. What about promoting active play, what kind of advice or support do you provide about this, if any? (Prompt: tummy time, floor play, limiting the use of constraints)
4. What would help you in promoting active play to parents and limiting TV and other media?

## 5.4 Opinion on use of websites and apps

*I would now like to ask your opinion on use of websites and smart phone apps for parent education purposes.*

1. What is your view about referring parents to websites or smartphone apps for information on healthy eating and active play? (Prompt: Is this something that you currently do? Why/why not?)
2. What features, if any, would you like to see available on a website or smartphone app about infant feeding and active play? (Prompt: fact sheets/resources, tracking and monitoring devices, videos from professionals/mums etc.)

# 6. Obesity Prevention in children

*Finally, I would like to hear more about your experiences and perspectives concerning overweight and obesity in children. To start with:*

1. How common a problem is it in the families (both the children and the parents) that you see?
2. What do you think the causes are? Is overweight and obesity preventable?
3. What’s the scope, if any, in your daily practice, to identify infants or children who might be at risk of becoming ‘overweight’?
4. Is it something that you try to do?

If YES 🡪 How do you identify children at risk of overweight? e.g. growth charts/BMI charts.

IF NO 🡪 I‘d like to hear more about your perspective. Is it an appropriate aspect of the CFHN role, for example…?

*Finally, just to wrap this up, could you briefly tell me about any instances when you initiated an intervention with parents whose infant/young child had gained weight too quickly, in your opinion?*

IF YES 🡪

1. What were the circumstances surrounding the situation?
2. What approach or strategies were used to address the situation?
3. Did you consider referring the family to another health practitioner – e.g. a dietitian?
4. How did it turn out? e.g. Did child lose weight as intended? How did parents respond? Was there any information or support you needed that wasn’t available to you at the time?
5. What lessons, if any, emerged from this experience?

IF NO 🡪 Thank you for your assistance, etc.
