# Supplementary material for: Promoting healthy weight for all young children: a mixed methods study of child and family health nurses’ perceptions of barriers and how to overcome them
Source: BMC Nurs. 2020 Sep 14;19:84. doi: 10.1186/s12912-020-00477-z (PMC7488672; doi:10.1186/s12912-020-00477-z)
Supplement: Supplementary file 3 — Additional file 3. [file 12912_2020_477_MOESM3_ESM.docx]

Child and Family Health Nurse survey demographics

**CFHN age**

|  | Frequency | % |
| --- | --- | --- |
| 20-29 years | 1 | 1.1 |
| 30-39 years | 13 | 14.4 |
| 40-49 years | 29 | 32.2 |
| 50-59 years | 36 | 40.0 |
| 60 years or over | 9 | 10.0 |
| Missing data | 2 | 2.2 |

**Years of experience as CFHN**

|  | Frequency | % |
| --- | --- | --- |
| Less than 5 years | 17 | 18.9 |
| 5-10 years | 24 | 26.7 |
| 11-15 years | 17 | 18.9 |
| More than 15 years | 30 | 33.3 |
| Missing data | 2 | 2.2 |

Child and Family Health Nurse access to infant and child health resources

| **Resource** | **Do you have easy access to the following? (n, %)** | |
| --- | --- | --- |
|  | Yes | No |
| Standard growth charts for infants 0-2 years | 89 (98.9) | 1 (1.1) |
| Body mass index (BMI) percentile chart for children aged 2-18 years | 84 (93.3) | 6 (6.7) |
| Educational materials for parents on infant feeding | 88 (97.8) | 2 (2.2) |
| Educational materials for parents on healthy eating for toddlers and pre-schoolers | 85 (94.4) | 5 (5.6) |
| Educational materials for parents on promoting active play in young children | 78 (86.7) | 12 (13.3) |
| Educational materials for parents on limiting sedentary activities (eg. TV watching) | 55 (61.1) | 35 (38.9) |
| Educational materials for parents on sleep and settling techniques for infants | 87 (96.7) | 3 (3.3) |

Child and Family Health Nurse views on promoting healthy weight gain in infants and young children

| **Statement** | Total responses | Response to statement (n, %) | |
| --- | --- | --- | --- |
|  |  | Overall agree^a^ | Overall disagree^b^ |
| Fatness or accelerated weight gain in infancy is NOT related to the development of overweight in childhood | 86 | 13 (15.1) | 73 (84.9) |
| It is easy to identify overweight infants and young children just by looking at them | 87 | 17 (19.5) | 70 (80.5) |
| It is easy to identify infants and young children who are at risk of becoming overweight | 87 | 47 (54.0) | 40 (46.0) |
| Providing advice to parents on infant feeding is an important part of my role | 88 | 88 (100) | 0 |
| Talking to parents about healthy infant feeding practices fits easily into my routine consultations | 88 | 88 (100) | 0 |
| I feel uncomfortable raising the issue of infants' and young children's weight with parents | 85 | 30 (35.3) | 55 (64.7) |
| For most parents, my advice and support does little to promote the adoption of a healthy lifestyle for the whole family | 84 | 21 (24.7) | 64 (75.3) |
| Providing advice on healthy eating and physical activity for the whole family is valued as an important part of my role by my managers | 86 | 8 (93.0) | 6 (7.0) |
| Some parents react negatively to me raising the issues of their child's weight | 88 | 58 (65.9) | 30 (34.1) |
| I have sufficient time to properly address healthy lifestyle behaviours with families with young children | 88 | 45 (51.1) | 43 (48.9) |
| Providing advice on healthy lifestyle behaviours for the whole family is an important part of my role | 86 | 82 (95.3) | 4 (4.7) |
| Parents I see are generally not interested in discussing the development of healthy lifestyle habits for their children | 87 | 16 (18.4) | 71 (81.6) |
| I find it professionally rewarding to address healthy lifestyle behaviours with families of young children | 86 | 81 (94.2) | 5 (5.8) |

^a^Overall agree = summation of ‘Agree’ and ‘Strongly agree’ responses.

^b^Overall disagree = summation of ‘Disagree’ and ‘Strongly disagree’ responses.

Highlighted statements are referenced in the manuscript.

Child and Family Health Nurse confidence in activities undertaken in infant and young child consultations

| **Activity** | Total responses | Confidence (n, %) | |
| --- | --- | --- | --- |
|  |  | Lower confidence^a^ | Higher confidence^b^ |
| Measuring infants' height & weight & plotting on a growth chart | 87 | 0 | 87 (100) |
| Calculating BMI for children 2 years & older & plotting on a BMI percentile chart | 87 | 11 (12.6) | 76 (87.4) |
| Identifying infants and young children who are at risk of overweight or obesity | 87 | 14 (16.1) | 73 (83.9) |
| Providing breastfeeding advice and support | 86 | 6 (7.0) | 80 (93.0) |
| Providing advice on correct formula preparation | 86 | 3 (3.5) | 83 (96.5) |
| Providing advice on sleeping and settling techniques for infants | 88 | 8 (9.1) | 80 (90.9) |
| Providing advice to parents regarding when to introduce solid foods to infants | 88 | 0 | 88 (100) |
| Providing advice to parents regarding how to introduce solid foods to infants | 88 | 1 (1.1) | 87 (98.9) |
| Talking to parents about eating their meals with their children | 86 | 2 (2.3) | 84 (97.7) |
| Talking to parents about limiting children's intake of sweetened drinks (eg. juice and soft drinks) | 86 | 0 | 86 (100) |
| * Talking to parents about offering water as the child’s main drink (after 12 months of age) | 45 | 0 | 45 (100) |
| * Talking to parents about limiting TV or other screen based activities | 44 | 2 (4.5) | 42 (95.5) |
| * Talking to parents about increasing active play for young children | 45 | 0 | 45 (100) |
| * Talking to parents about increasing their children’s fruit and vegetable intake | 45 | 0 | 45 (100) |
| * Talking to parents about limiting children’s intake of high sugar and/or high fat foods (eg. cakes, biscuits, lollies, chips, takeaway foods, etc.) | 45 | 0 | 45 (100) |

^a^Lower confidence = summation of ‘Not at all confident’ and ‘Somewhat confident’ responses.

^b^Higher confidence = summation of ‘Very confident’ and ‘Extremely confident’ responses.

Highlighted statements are referenced in the manuscript.

Questions marked with an asterisk (*) were accidentally omitted from the online REDCap survey, resulting in 45 missing responses – however, >95% of nurses who responded to these questions responded in the ‘higher confidence’ summated category.
